# FDG-PET and PET/CT - Part II

**DOI:** 10.4103/0971-3026.38504

**Published:** 2008-02

**Authors:** Amol M Takalkar, Ghassan El-Haddad, David L Lilien

**Affiliations:** * PET Imaging Center, Biomedical Research Foundation of Northwest Louisiana, Shreveport, LA, USA; ^ Department of Radiology, Nuclear Medicine Section, Louisiana State University Health Sciences Center - Shreveport, Shreveport, LA USA

**Keywords:** FDG, PET, PET/CT

## Introduction

The high diagnostic accuracy of FDG-PET and PET/CT has been well documented for several neoplasms, with a consequent rapid increase in the utilization and acceptance of PET imaging in the clinical setting. As reported earlier,[1] initial work with PET that started in the 1980s had already established the value of FDG-PET in neurologic and cardiac applications.[2] FDG-PET became a clinical reality only when its importance in the management of several common malignancies was demonstrated and documented by many investigators in the early and late 1990s. The *Warburg Effect* (increased aerobic glycolysis in cancer cells) is the basic principle underlying the value of FDG-PET imaging in neoplasms.[3] FDG-PET is now routinely utilized in the evaluation of several neoplasms like lymphoma, melanoma, lung cancer, breast cancer, colorectal cancer, to name a few. Its value in several other neoplasms is being explored in the United States of America (US) with the establishment of a National Oncologic PET registry (NOPR). The introduction of dual modality PET/CT imaging in the late 1990s and early part of this century has added more strength to this powerful imaging modality. Today, FDG-PET or PET/CT imaging has become an integral component in the evaluation of numerous neoplasms and has been shown to have a tremendous clinical impact on the management of a significant number of cancer patients. In addition to FDG, other PET radiopharmaceuticals are also being developed for cancer imaging.

This article reviews the current clinical applications of FDG-PET and PET/CT in certain malignancies, specifically head and neck cancer, lung cancer, lymphoma and melanoma. Thyroid neoplasms are included in the head and neck cancer section and pleural malignancies are discussed in the lung cancer section.

## FDG-PET and PET/CT in oncology

Anatomic imaging modalities like CT scan and MRI have superb structural resolution that is continuously improving with technological innovations. However, these imaging modalities rely significantly (although not exclusively) on size and shape of lesions, in addition to indirect findings such as edema, to differentiate benign from malignant disease. Therefore, disease manifestations that occur at the molecular and cellular level, before manifesting as structural changes, are frequently not detected with these modalities. FDG-PET has the capability to depict abnormal metabolic activity before any anatomic change occurs. This is particularly important in patients who undergo therapeutic interventions such as radiotherapy and chemotherapy, after which structural changes often lag behind metabolic changes, for a significant interval of time. In fact, some lesions may never shrink to normal size or disappear completely in spite of adequate treatment. Functional or metabolic imaging with FDG-PET complements structural imaging and helps overcome some of these major shortcomings. As discussed in the previous article in this series,[1] FDG accumulates at a higher rate in cancer cells and remains metabolically trapped in these cells. This phenomenon is the underlying basis for FDG-PET imaging in oncology. FDG-PET has been shown to have a high sensitivity and negative predictive value for the detection of malignancy. Hence in several neoplasms (with notable exceptions), FDG-PET imaging plays a critical role in initial diagnosis, staging, assessing response to therapy, restzaging and assessing recurrence. The common neoplasms that have been approved by Centers for Medicare and Medicaid Services (CMS) in the US to be evaluated by FDG-PET include lymphoma, melanoma, head and neck cancer, lung cancer (except small cell lung cancer), breast cancer, esophageal cancer, colorectal cancer, cervical cancer and thyroid cancer (recurrence). However, FDG-PET has also been shown to be useful in several other neoplasms, such as gastric cancer, uterine and ovarian cancers, testicular cancer, soft tissue sarcomas and others. It is also known that a few cancers like prostate cancer and hepatocellular carcinoma do not show increased FDG uptake in a significant portion of patients; and other neoplasms, including pancreatic cancer, are affected by the low specificity of FDG uptake. Renal cancers are difficult to assess on FDG-PET imaging due to physiologic renal excretion of FDG. In May 2006, a National Oncologic PET Registry (NOPR) was established in collaboration with the American College of Radiology Imaging Network (ACRIN), the American College of Radiology (ACR) and the Academy of Molecular Imaging (AMI), in response to the proposal by Centers for Medicare and Medicaid Services (CMS) to expand coverage of FDG-PET studies to indications not currently eligible for Medicare reimbursement. The NOPR received inputs from and is endorsed by, the ACR, the American Society of Clinical Oncology (ASCO) and the Society of Nuclear Medicine (SNM) and will provide important data regarding the utility of FDG-PET in several neoplasms not routinely evaluated with FDG-PET imaging.

### Head and neck cancer

Malignancies involving the head and neck region have generally been declining in incidence, but about 68,000 new cases are still estimated to occur in the US in 2007.[4] These are usually associated with alcohol and tobacco use; and other factors like poor nutrition and oral hygiene, immunity disorders, (human papilloma virus) HPV infections and occupational exposure. Head and neck cancers include malignancies of the lip and oral cavity; nasopharynx oropharynx and hypopharynx; larynx; ear, paranasal sinuses and salivary glands. Thyroid neoplasms will be discussed separately in the next section in view of their completely different behavior and consequent therapeutic implications, as compared to the rest of the cancers in the head and neck. In addition, lymphoma and other malignancies like melanoma may also involve the head and neck region, but will be discussed in their respective sections.

The majority of head and neck cancers tend to be squamous cell carcinomas (HNSCC) that show intense FDG uptake.[5] FDG-PET imaging plays an important role in diagnosis (especially in the setting of metastasis from an unknown primary site), staging, restaging (crucial for assessing residual/ recurrent disease) and monitoring response to therapy. The advent of combined PET/CT imaging has further strengthened the role of this imaging modality in the overall workup of head and neck cancers because of the intricate anatomy in this region.

The diagnosis of head and neck cancer is usually established on the basis of directed biopsies during an endoscopic evaluation (laryngoscopy, nasopharyngoscopy or panendoscopy). Head and neck cancers are staged as per the Tumor, Node and Metastasis (TNM) staging criteria. Along with clinical evaluation with direct visualization, contrast-enhanced CT and MRI are extremely useful for optimal assessment of the ‘T’ stage as they provide further structural information as regards tumor extension and involvement of adjacent structures. FDG-PET imaging lacks the spatial resolution for providing such exquisite structural details. Moreover, small lesions with low tumor volumes, superficial lesions (<4 mm deep) and very low grade tumors (including carcinoma *in situ*) are not readily visualized on FDG-PET imaging.[6] However, FDG-PET plays an important role in the detection of locoregional metastatic lymphadenopathy and distant metastatic disease.

**Figure 1:**
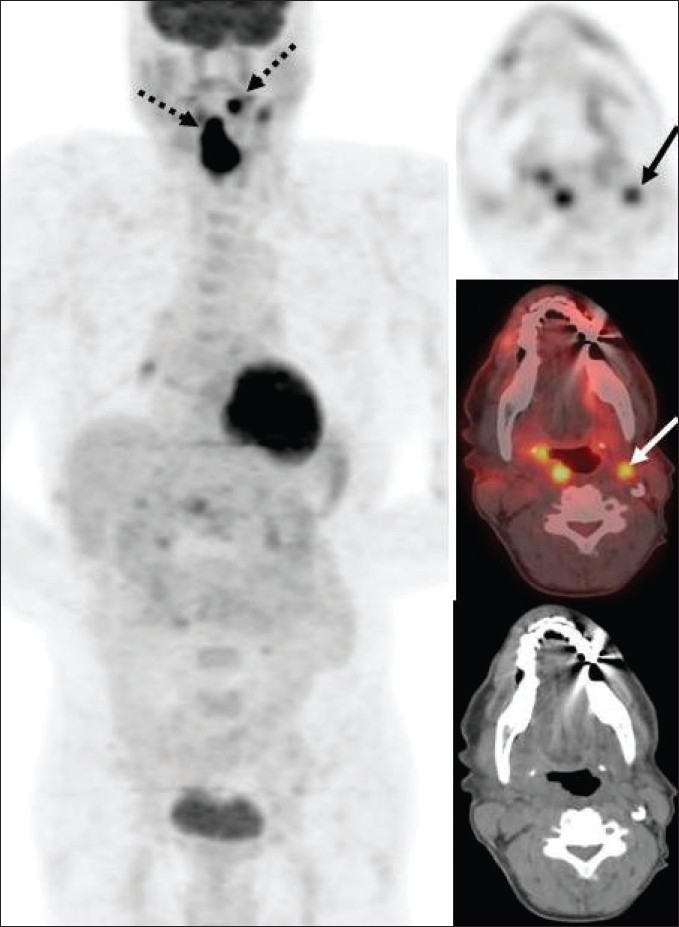
Axial PET (top right), fused PET/CT (middle right) and CT scan (bottom right) images at the submandibular region level, with an MIP image (on left), from a PET study of a 66-year-old man recently diagnosed to have squamous cell carcinoma of the oropharynx. Clinical and CT evaluation staged the patient as T2, N0, M0. The PET scan reveals the large primary lesion in the oropharynx to be intensely FDG avid (dotted arrow) with another left oral cavity lesion (dotted arrow) and a left submandibular nodal focus (arrows). The patient had no visible lesion in the left oral cavity at the time of initial examination but subsequently developed an ulcerative lesion in the left lingual sulcus. The submandibular nodal focus localized to an 8-mm node on the CT scan and was not considered enlarged per CT criteria. The patient also had a moderate right hilar focus (seen on the MIP image) and subsequently developed into a non-small cell lung cancer a year later

**Figure 2:**
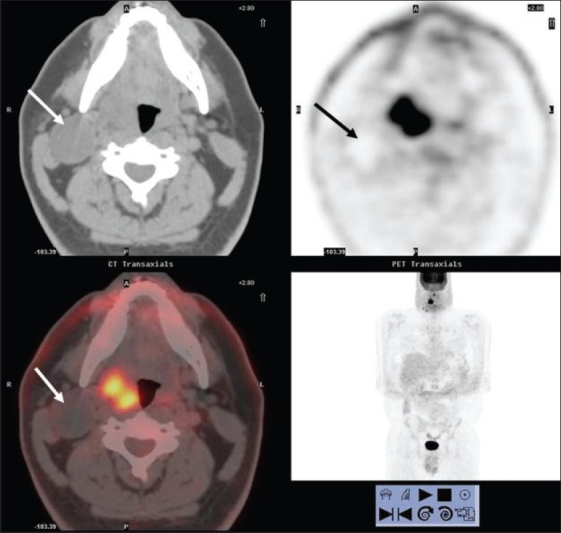
Axial PET (top right), CT scan (top left) and fused PET/CT (bottom left) images at the submandibular region level, with an MIP image (bottom right), from a PET study of a 44-year-old man with right tonsillar cancer that is intensely FDG avid. However, an enlarged hypodense nodal mass (white arrows) posterior to the angle of the right mandible is completely ‘cold’ on PET but clinically was hard and consistent with metastasis (also confirmed on subsequent neck dissection)

Only about 10-15% of head and neck cancers have distant metastases at the time of initial presentation and hence therapy is mainly determined by ‘T’ and ‘N’ status in a majority of patients. Traditional anatomical evaluation of nodal involvement in the head and neck region is suboptimal, since nodes may be enlarged as a result of infection/ inflammation (this is not uncommon in the head and neck region) and normal-sized nodes may frequently be involved with metastatic disease, leading to inaccurate upstaging or downstaging of the disease with conventional imaging methods. FDG-PET (and especially PET/ CT) imaging has had a tremendous impact in improving the nodal staging of head and neck cancers. Numerous studies have documented the high sensitivity (87%), specificity (94-95%) and accuracy of FDG-PET imaging in assessing nodal involvement in head and neck cancers.[6–10] FDG-PET imaging frequently detects metastatic disease in normal-sized lymph nodes [Figure 1]. However, caution is recommended in N0 disease per PET as micrometastases cannot be detected by FDG-PET imaging and hence the management of such patients should not solely be determined by FDG-PET findings[11] and other techniques like sentinel node mapping or surgical neck dissection should be employed for optimal ‘N’ staging in such patients. Also, sometimes malignant cervical nodes with large extensive central necrosis can be falsely negative on FDG-PET, with only mild FDG uptake at the periphery or no uptake at all [Figure 2].

**Figure 3:**
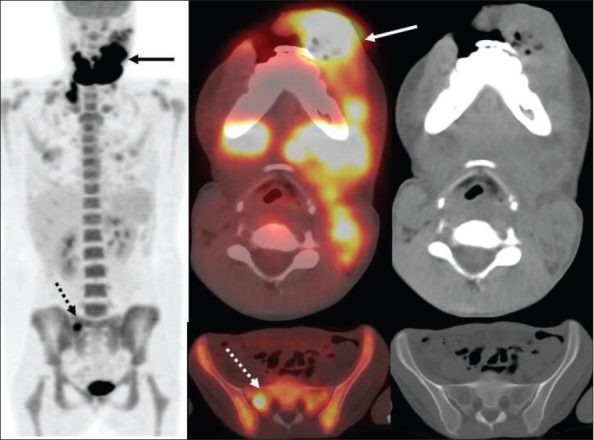
Fused PET/CT (middle) and CT scan (right) images at the level of the lip (top) and pelvis (bottom), with an MIP image (on left), from a PET study of a 34-year-old lady with locally extensive invasive squamous cell carcinoma of the lip. The PET scan not only shows extremely locally advanced lip neoplasm (arrows) but also extensive loco-regional nodal metastases along with multiple pulmonary metastases, bone and bone marrow metastases (dotted arrow points to an intense right sacral ala lesion), thus significantly upstaging the disease stage

**Figure 4:**
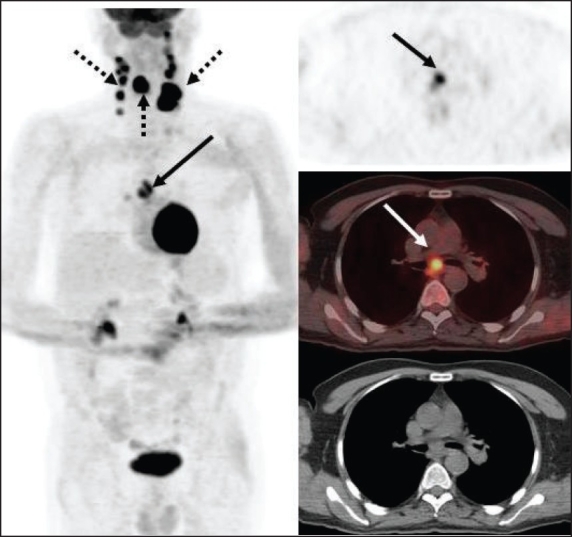
PET (top right), fused PET/CT (middle right) and CT scan (bottom right) images at the subcarinal level, with an MIP image (on left), from a PET study of a 66-year-old man recently diagnosed with T3, N2c, Mx invasive squamous cell carcinoma of the supraglottis. The PET scan not only confirms the large primary lesion with bilateral extensive cervical metastatic adenopathy (dotted arrows) but also shows an unsuspected intense subcarinal nodal metastasis in the mediastinum (arrows), subsequently confirmed on biopsy, thus upstaging the patient to stage IV and excluding curative therapy

In addition to improved nodal staging, the most important added value of FDG-PET imaging is in the detection of unsuspected distant metastases [Figure 3] that can lead to dramatic changes in patient management. As seen in [Figure 4], unsuspected mediastinal nodal metastases (subsequently proven by mediastinoscopy and biopsy) detected on FDG-PET imaging in this patient with head and neck cancer altered the management options significantly and prevented futile attempts at curative treatment. Incorporation of FDG-PET imaging in the initial staging changes management in about 10% of patients suspected to have advanced HNSCC.[12]

**Figure 5:**
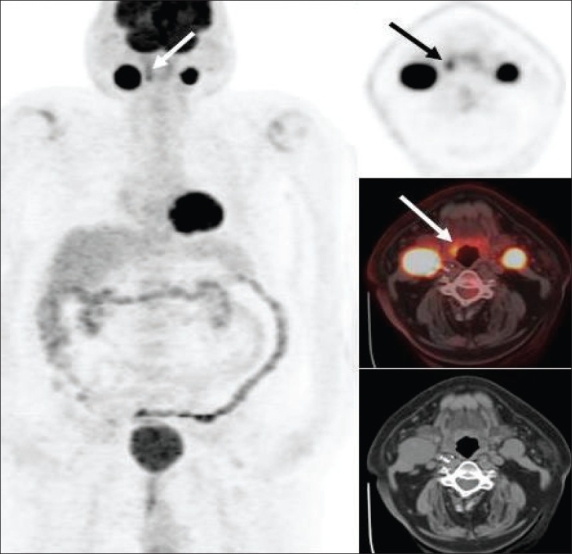
PET (top right), fused PET/CT (middle right) and CT scan (bottom right) images at the level of the tonsils, with an MIP image (on left), from a PET study of a 70-year-old man presenting with a right neck mass. CT scan of the neck revealed bilateral enlarged neck nodes that showed squamous cell carcinoma on biopsy. However, the primary lesion remained unknown after clinical, CT scan and panedoscopic evaluation. The PET scan shows asymmetric tonsillar uptake with slightly more intense uptake in the right tonsil (arrows), as compared to the left. Subsequent biopsy confirmed a grade 2 invasive right tonsillar squamous cell carcinoma

**Figure 6 (A, B):**
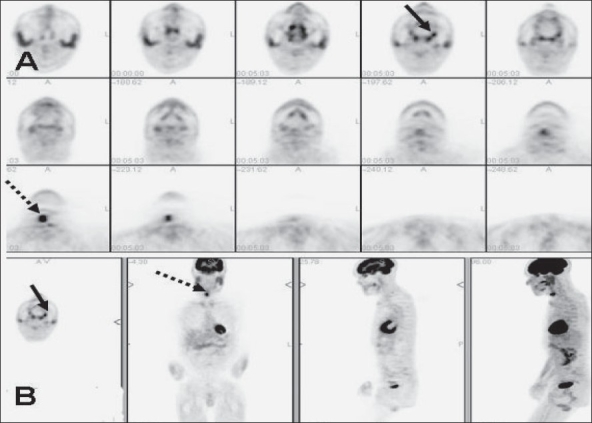
PET images of a 67-year-old man presenting with hoarseness and found to have a right vocal cord lesion. Axial images (A) from the skull base to the thoracic inlet and a set (B) of triaxial image at the level of oral cavity, along with coronal and sagittal images and a sagittal MIP image show not only an intense right vocal cord lesion (dotted arrow) but also a focus in the left retromolar area (arrow). Biopsy revealed a T1 left retromolar trigone squamous cell carcinoma and a T2b right vocal cord carcinoma

Due to its high sensitivity and the ability to perform whole-body scanning, FDG-PET is used in the evaluation of patients presenting with malignant cervical lymphadenopathy without evidence of a primary neoplastic lesion on conventional investigations, including CT scan / MRI and panendoscopy with directed biopsies. In such cases, FDG-PET and PET/CT can help identify the primary malignant lesion [Figure 5] in about 25% of cases.[13,14] At our center, a retrospective chart review revealed that using FDG-PET, we were able to identify the primary tumor in 61% of patients (14 of 23 cases) who presented with this scenario[15] with a very high negative predictive value (89%). Our higher rate of detection of the occult primary lesion may be attributable to several factors, including rigorous scan protocol (valium pre-treatment oral and intravenous hydration, 90-min delayed imaging), experienced readers, interpretations tuned to increase sensitivity (but not specificity) in this situation [Figure 5] and a relatively small number of patients in this study. Since head and neck cancer patients are also prone to have synchronous neoplasms either in the head and neck region or elsewhere in the body, as well as subsequent development of second primaries (most frequently in the lung), FDG-PET imaging plays an important role in diagnosing these unexpected lesions[16] [Figure 6].

FDG-PET imaging is useful in therapy planning for patients undergoing radiation therapy with a curative or palliative intent or as neoadjuvant therapy. The increasing implementation of intensity-modulated radiation therapy (IMRT) is well complemented by the additional functional/metabolic information provided by the FDG imaging as it allows the delivery of maximal radiation dose to the most metabolically active areas of the tumor and more complete inclusion of loco-regional disease with sparing of the uninvolved areas. FDG-PET is very helpful in evaluating the cervical nodal chains in patients with advanced-stage head and neck cancer, since the presence of contralateral cervical lymphadenopathy will necessitate the inclusion of both sides of the neck in the radiation therapy field.

**Figure 7:**
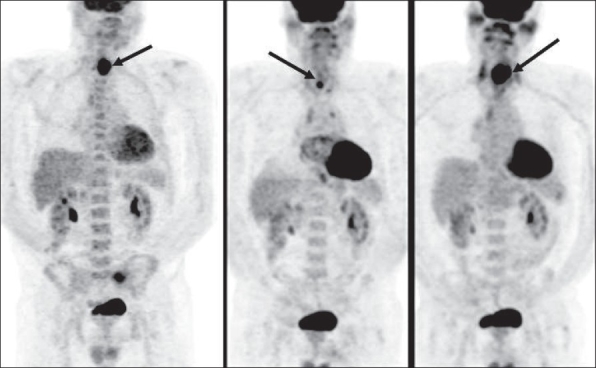
MIP images from three different PET studies of a 65-yearold man with laryngeal cancer, detected incidentally on a PET study (arrow in the left image) for a right lung mass (which was not FDG avid and was proven to be benign). A repeat PET study (middle image) 3 months after completing concurrent chemoradiation therapy shows significant but incomplete response with some residual disease (arrow). However, biopsy failed to reveal residual disease and the patient was followed routinely without additional therapy. Five months later, the patient required emergent admission for a tracheostomy for shortness of breath. Vocal cord biopsy revealed malignant disease and a subsequent PET study (right image) shows significant progression of disease (arrow).

In addition to the above, FDG-PET imaging is very useful in assessing response to therapy and in restaging of head and neck cancers.[17,18] Following surgery or radiation therapy, it is extremely difficult to assess the neck with conventional imaging modalities like CT scan/MRI due to inflammatory changes, fibrosis, edema and alteration of normal structures. Determining whether residual neoplasm is present in the postsurgical/ post-radiated tumor bed is a daunting task. Compared to conventional radiological examination, FDG-PET has a better diagnostic accuracy in the assessment of residual or recurrent malignant disease in the post-therapeutic neck, including avoidance of unnecessary planned surgery in patients with a negative PET study.[19] Indeed, at our center, we have on several occasions seen intense residual disease after therapy on a post-treatment PET scan with a negative biopsy, which has then progressed to a larger recurrent lesion a few months later [Figure 7]. A negative tissue biopsy after a strongly positive post-treatment PET scan can be caused by a sampling error and warrants a closer follow-up rather than routine surveillance. A decrease in the intensity of uptake on a follow-up scan confirms a false-positive post-treatment PET scan, usually due to inflammatory changes. However, persistence of a focally intense lesion or increase in the intensity of uptake warrants invasive evaluation. The timing of post-treatment PET scan is very crucial, especially after radiation therapy. Although there are no specific recommendations, generally a 3-month interval after completing radiation therapy is felt to be adequate to assess response to therapy. However, if surgery is needed for therapy completion, it is preferable to perform the surgical intervention much sooner than 3 months after radiation since more delay makes surgical dissection difficult due to post-radiation fibrosis. Hence at our center, we image such patients about 6-8 weeks after completion of radiation therapy. The superior assessment of response to therapy with FDG-PET imaging has facilitated a more conservative approach in management. In patients undergoing combined chemo-radiation therapy with a complete response on the post-treatment FDG-PET scan, there is a trend towards a more watchful approach rather than proceeding with a completion neck dissection in spite of large nodes (>3 cm) at initial staging.

**Figure 8:**
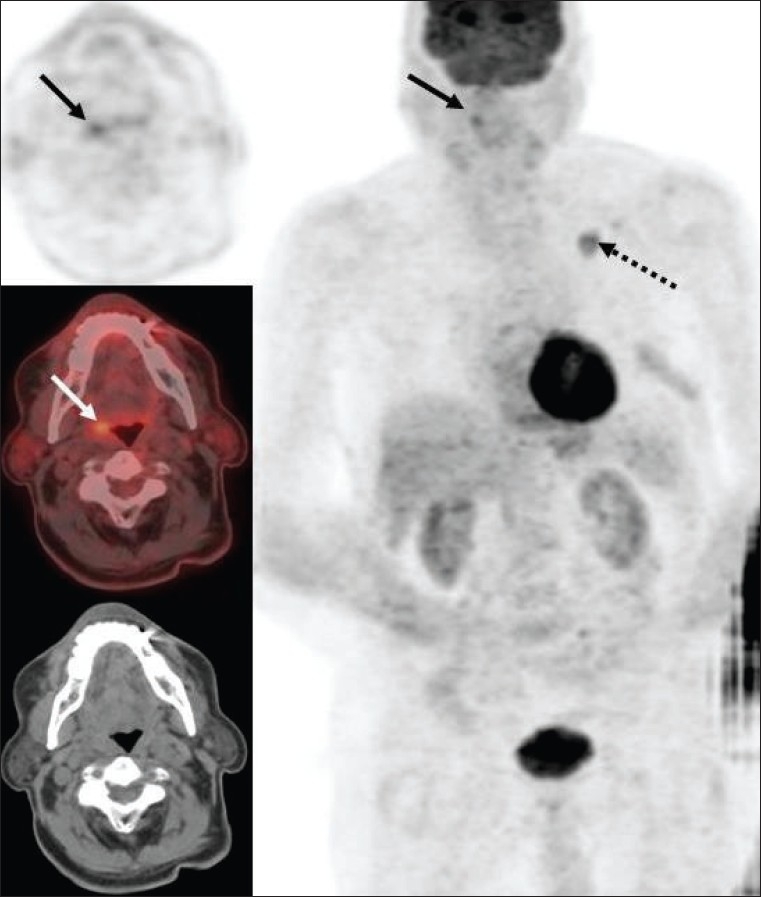
Axial PET (top left), fused PET/CT (middle left) and CT scan (bottom left) images at the level of the tonsils, with an MIP image (on right), from a PET study of an 80-year-old man with a left lung cancer that shows increased FDG uptake (dotted arrow). Incidentally noted is increased uptake in the right tonsil (arrows), which was found to be due to chronic inflammation

There are several limitations of FDG-PET imaging in the evaluation of head and neck cancer. Although it may detect tumors that may be missed by anatomic imaging (especially in the setting of an unknown primary), the suboptimal spatial resolution as compared to direct visualization with endoscopy and CT scan / MRI limits the evaluation of small and superficial lesions. Also, low-grade tumors may be missed on PET if there is significant intense physiologic FDG uptake in an adjacent structure. As described in the earlier article in this series,[1] a number of structures in the head and neck region show physiologic FDG uptake, which can sometimes be quite intense. However, physiologic FDG activity is usually symmetric and the added information from CT images in a dedicated PET/CT scan can further help to discern this uptake as benign/ physiologic. Asymmetric FDG uptake (especially in the tonsils) can cause problems with interpretation. In patients being evaluated for cancers other than in the head and neck region, asymmetric tonsillar activity [Figure 8] is frequently caused by tonsillar inflammation or lymphoid hyperplasia (unless it is strikingly intense, in which case it suggests an undetected second primary malignancy in the tonsil). In order to avoid physiologic muscle and brown fat activity that could potentially affect the interpretation of the study in the head and neck region, it is necessary to place the patient in a comfortable position and room temperature. In addition, benzodiazepines (diazepam is most commonly being used), help prevent brown fat activity. Chewing gum on one side in the mouth can give rise to unilateral intense FDG uptake in the muscles of mastication. Benign uptake in inflammatory/ infectious lesions, Warthin's tumor of the parotids (which can be intensely FDG-avid), radiation-induced inflammation and osteoradionecrosis need to be kept in mind. There may be variable uptake in the vocal cords caused either by talking/ snoring during the FDG uptake interval or due to unilateral vocal cord impairment or iatrogenic causes like Teflon injection.

### Thyroid malignancies

Approximately 33,500 new thyroid cancer cases are estimated in the US in 2007, with about 1,500 deaths.[4] Thyroid neoplasms are predominantly of follicular or para-follicular cell origin. Thyroid neoplasms of follicular cell origin include papillary carcinomas, which represent 80% of all thyroid neoplasms; follicular carcinomas (second most common); as well as the less common but more aggressive Hurthle cell (3-10%) and anaplastic carcinomas (1-2%). The para-follicular or neuroendocrine-derived calcitonin-producing C cells give rise to medullary thyroid carcinomas (MTCs), which represent 5-10% of thyroid neoplasms, a small portion of which may be associated with multiple endocrine neoplasm (MEN) syndromes. In addition, there are rare thyroid neoplasms such as primary thyroid lymphomas and sarcomas.

**Figure 9:**
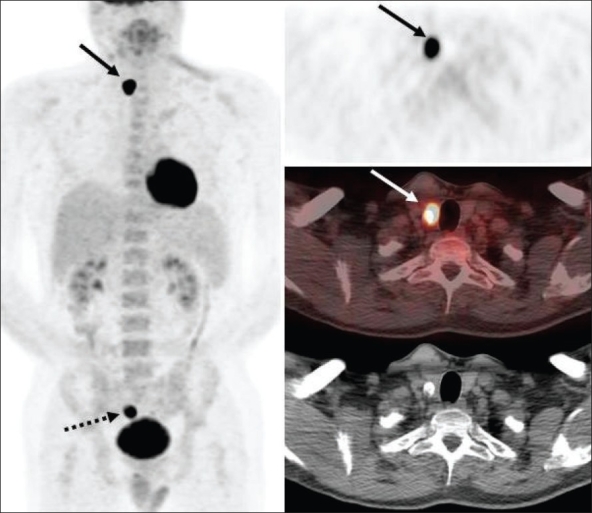
Axial PET (top right), fused PET/CT (middle right) and CT scan (bottom right) images at the level of the thyroid gland, with an MIP image (on left), from a PET study of a 42-year-old man, status post-surgical resection and neck dissection for a T2, N1, M0 left retromolar trigone mucoepidermoid carcinoma. The PET scan shows an incidental intense calcified right thyroid lobe lesion (arrow), which was proven to be a Hurthle cell carcinoma on resection. Another incidental intense focus is seen in the pelvis (dotted arrow), which was found to be a tubular adenoma in a colonic polyp.

**Figure 10:**
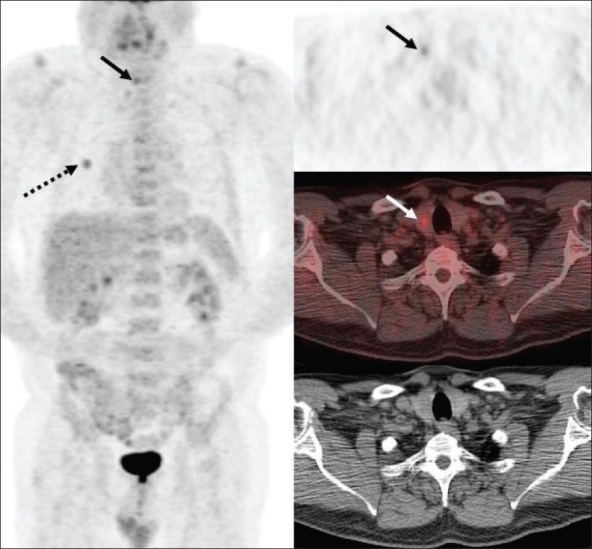
Axial PET (top right), fused PET/CT (middle right) and CT scan (bottom right) images at the level of the thyroid gland, with an MIP image (on left), from a PET study of a 65-year-old man with a right lung nodule. The PET scan shows increased uptake in the right lung nodule (dotted arrow). Also noted incidentally is a subtle right thyroid lobe abnormality (arrows), which was proven to be a well-differentiated thyroid carcinoma on further evaluation

Generally, thyroid neoplasms are diagnosed after workup for a painless thyroid nodule/ mass. Most malignant thyroid nodules show increased FDG uptake.[20] Usually, well-differentiated thyroid neoplasms tend to have lower FDG avidity (but excellent radio-iodine avidity) and poorly differentiated thyroid malignancies like Hurthle cell [Figure 9] and anaplastic carcinomas tend to show intense FDG uptake (but poor iodine accumulation). In fact, the highest published standard uptake value SUV on a PET scan (SUV_max_: 125.1, SUV_avg_: 66.2) in the literature has been in an anaplastic thyroid cancer.[21] Hurthle cell thyroid cancer has generally low avidity for radio-iodine and FDG accumulation is usually very intense, revealing disease not detected by other imaging methods in about 50% of cases.[22] False negative FDG-PET exams can be seen in patients with iodine-avid Hurthle cell cancers. However, there is no defined role for FDG-PET imaging in the initial diagnosis and staging of thyroid malignancies. More frequently, there is incidental detection of focally intense lesions in the thyroid gland on scans performed to evaluate other malignant conditions. Such focal intense thyroid ‘incidentalomas’ warrant further evaluation, since about 25-30% of these turn out to be thyroid carcinomas.[23,24] Sometimes, these ‘incidentalomas’ may be quite subtle [as in Figure 10], but still warrant evaluation since they can be well-differentiated thyroid carcinomas (as was proven in this case).

**Figure 11 (A-B):**
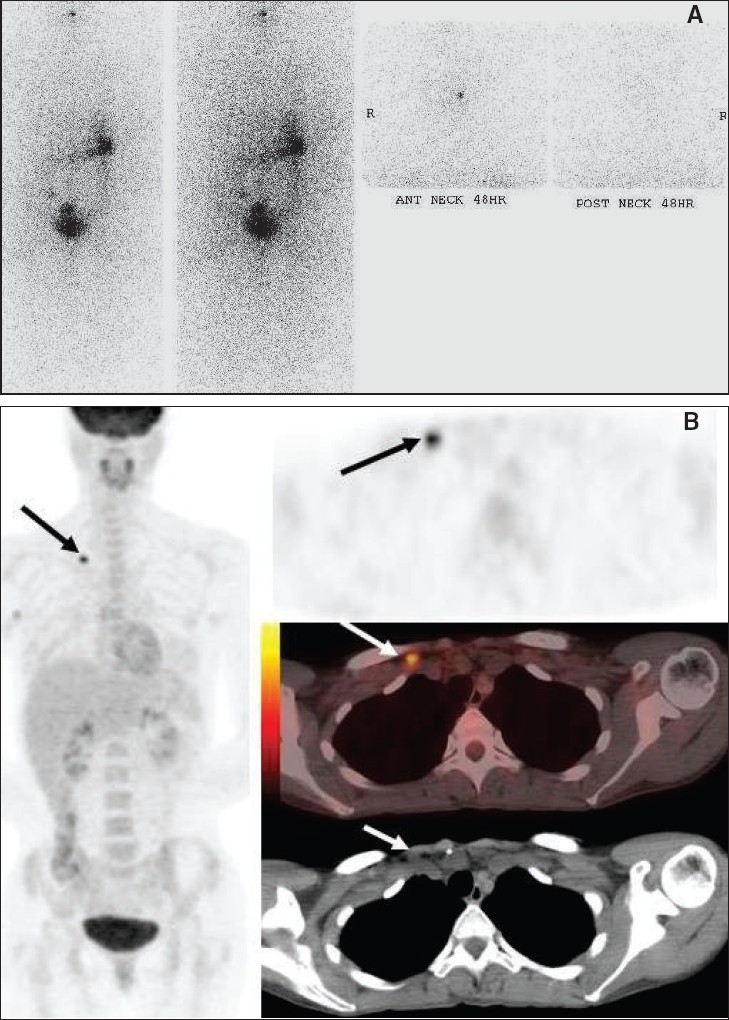
A 32-year-old lady with a history of pT4a, pN1b, Mx papillary thyroid cancer treated with surgery followed by radio-ablation with 200 mCi of I-131 with no I-131 avid metastases on the 7-day post-treatment scan. She continued to have elevated thyroglobulin levels; but physical examination, USG, CT scan and repeat imaging with I-131 whole-body scan (A) failed to reveal evidence of residual/recurrent disease. Subsequent PET study (B) with axial PET (top right), fused PET/CT (middle right) and CT scan (bottom right) images at the level of the supraclavicular fossa, with an MIP image (on left), showed an intense right supraclavicular node (arrows), measuring 1.0 cm in diameter, which was proven to be metastatic thyroid cancer

Except for anaplastic carcinomas, which are very aggressive rapidly progressing tumors, follicular thyroid neoplasms are treated with thyroidectomy followed by radio-iodine therapy with I-131. Follow-up usually consists of physical examination, serum thyroglobulin (Sr. Tg) levels and I-131 or I-123 whole-body scans to detect recurrence. Whole-body iodine imaging may be negative in 10-15% of patients with elevated Tg levels and clinical suspicion for disease recurrence, which can be a result of small volume disease or tumor dedifferentiation.[25] In this scenario, FDG-PET imaging is believed to be useful in detecting recurrent disease in about 70-90% of cases, especially if the Sr. Tg levels are more than 10 μg/dl.[24] However, the effect of TSH levels on the ability of FDG-PET to detect disease recurrence is still not clearly defined. Recent research suggests that elevated TSH levels (more from exogenous stimulation with recombinant TSH than with endogenous TSH stimulation via thyroid hormone withdrawal) may improve lesion detectability on FDG-PET imaging.[26–28] Such intensely FDG-avid lesions [Figure 11] suggest dedifferentiation of the tumor (possibly causing lack of iodine avidity) and may also indicate the need for additional surgical resection or radiation therapy. FDG imaging is an independent predictor of survival in patients with recurrent thyroid cancer and its prognosis is worse in patients with positive FDG scans.[29]

MTCs behave differently from follicular thyroid carcinomas. Instead of secreting thyroid hormones, they secrete calcitonin as well as carcinoembryonic antigen (CEA). Primary treatment is surgical resection in the form of thyroidectomy. Radio-iodine therapy or radio-iodine scanning for detection of recurrence is of no use in these patients. MTCs are followed by assessing serum calcitonin and CEA levels. In the setting of elevated serum calcitonin and/ or CEA levels, FDG-PET imaging is probably the most accurate modality to detect recurrent disease with a sensitivity and specificity of around 80% (sensitivity ranging from 40-95% in various studies).[30–32] A recent paper from Memorial Sloan Kettering Center in New York[30] reported that in patients with Sr. calcitonin levels <500 pg/ml, FDG-PET has limited sensitivity in detecting recurrence and achieves meaningful sensitivity only when the levels are more than 1000 pg/ml.

### Lung malignancies

**Figure 12:**
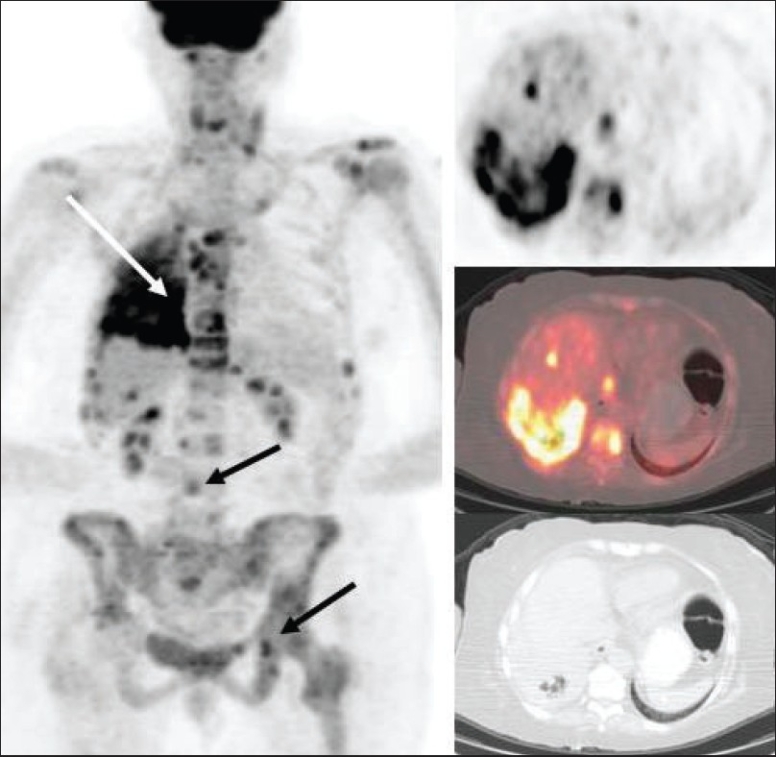
Axial PET (top right), fused PET/CT (middle right) and CT scan (bottom right) images at the level of the lung bases, with an MIP image (on left), from a PET scan of a 62-year-old lady with bronchoalveolar carcinoma of the right lung. PET scan shows intense uptake in the right lung base lesion (white arrow) and widespread metastases (black arrows).

In the United States, lung cancer remains the leading cause of cancer mortality for both men and women.[4] Broadly, lung cancers are classified into two major groups: small cell and non-small cell. Non-small cell lung carcinoma (NSCLC) is the more common type (75-80%) and includes several histological subtypes like squamous, adenocarcinoma, large cell, carcinoid, etc. Small cell lung carcinoma (SCLC) is less frequent (20-25%) but is frequently metastatic at the time of diagnosis and has different staging, therapeutic and prognostic implications. There is increased FDG activity in most lung cancers, but bronchoalveolar carcinomas (BAC) and lung carcinoids are traditionally considered non-FDG-avid. However, BAC and atypical lung carcinoids have been known to show increased FDG uptake on occasion [Figure 12].

**Figure 13:**
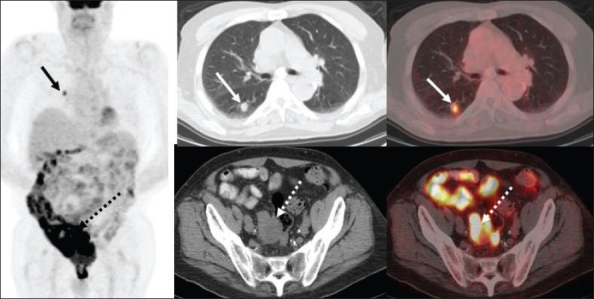
Axial CT and fused PET/CT images at the subcarinal level (top middle and right) and through the pelvis (bottom middle and right), with an MIP image (on left), from a PET study of a 67-year-old man being evaluated for a solitary lung nodule. The right lung nodule shows intense FDG uptake (arrows) consistent with a malignant etiology. Also noted is significant FDG uptake in the cecum and rectosigmoid colon (dotted arrows), for which a colonoscopic evaluation was recommended. The patient was subsequently found to have a large ulcerated rectosigmoid colon adenocarcinoma with tubular adenomas and pan-diverticular disease elsewhere in the colon. After resecting the colon cancer, the solitary lung nodule was also resected and found to be metastatic.

A solitary pulmonary nodule (SPN) is a frequent indication for a PET scan. Although many incidentally detected SPNs tend to be benign, in a high-risk patient (e.g, history of smoking), they are more likely to be malignant. As many as 20-30% of lung neoplasms initially present as SPNs and about 10-30% of all malignant SPNs are metastases from extrathoracic neoplasms [Figure 13]. In view of the improved spatial resolution of CT scans, many more and smaller SPNs are routinely being detected. It is often a challenge to differentiate benign from malignant lesions. Although structural characteristics such as configuration, size, enhancement and calcification provide significant information, the majority of SPNs remain indeterminate on conventional imaging. Tissue diagnosis by biopsy or aspiration (bronchoscopic or CT-guided) or wedge resection although invasive is frequently necessary to confirm the true nature of the lesion. However, in high-risk patients with coexisting morbidities like severe chronic obstructive pulmonary disease (COPD), FDG-PET imaging plays an important role in the workup of these lesions.

**Figure 14:**
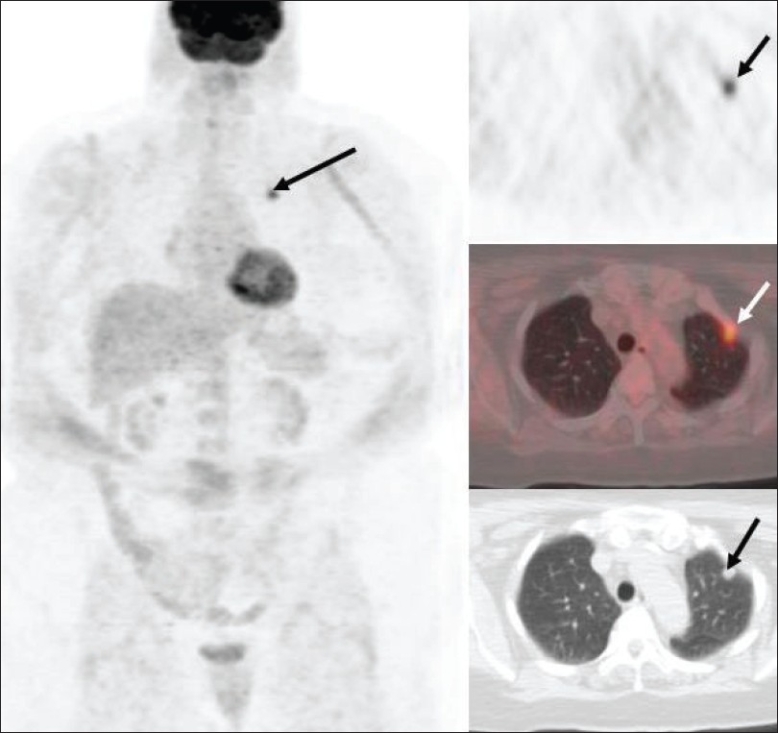
Axial PET (top right), fused PET/CT (middle right) and CT scan (bottom right) images at the level of the aortic arch, with an MIP image (on left), from a PET study of a 67-year-old patient with a new left upper lobe lung nodule. Intense FDG uptake in this nodule (arrow) is consistent with neoplastic disease unless proved otherwise. Tissue diagnosis is however necessary to confirm this and to exclude a false positive study

In general, any SPN with intense FDG uptake is considered to be of neoplastic etiology unless proved otherwise [Figure 14]. Although prior literature suggests an SUV cutoff of 2.5 (lesions with SUV >2.5 are considered malignant),[33–35] this is an oversimplification. Visual comparison of FDG uptake intensity with the mediastinal blood pool or liver activity is felt to be an equally reliable indicator of a neoplastic etiology. Another important consideration in the evaluation of SPNs with FDG-PET imaging is their size. Generally, modern PET and PET/CT scanners have a spatial resolution of about 6-8 mm and lesions less than 6 mm may not be adequately assessed; the intensity/ SUV may not be very high in such sub-centimeter lesions. Conversely, if a tiny lesion, supposedly below the spatial resolution for PET imaging, shows increased FDG activity, it is quite suspicious for a malignant etiology (even though the intensity may be mild).

**Figure 15:**
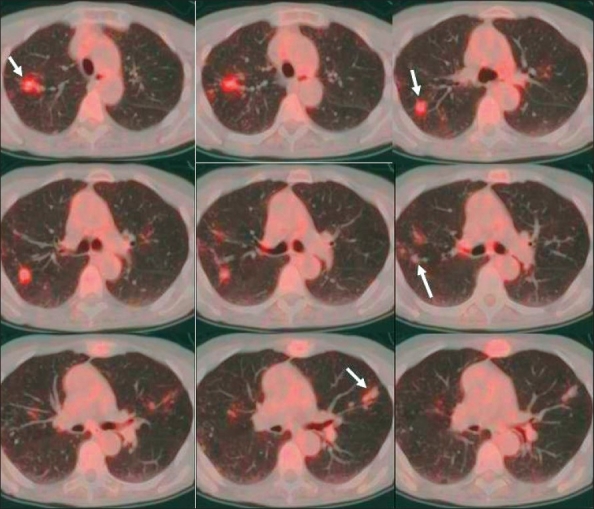
Fused axial, lung window, PET/CT images of the chest from a PET study of a 73-year-old patient with bilateral lung nodules. These generally show mild-to-moderate uptake (arrows), with more intense uptake in one of the right upper lobe nodules and with almost absent uptake in some of the left upper lobe nodules. Transbronchial biopsy was negative for malignancy and the patient was subsequently found to have Mycobacterium Avium-Complex (MAC) infection

**Figure 16:**
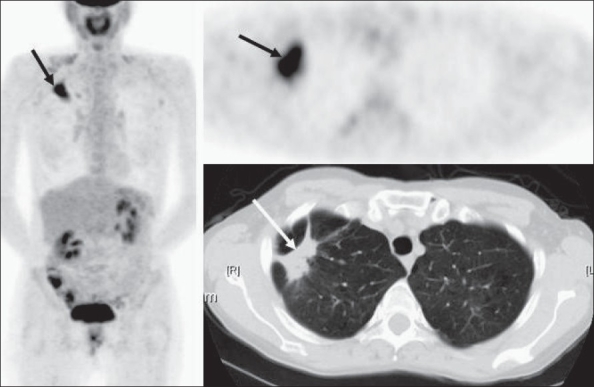
Axial PET (top right) and CT scan (bottom right) images at the level of the upper lungs, with an MIP image (on left), from a PET study of a 46-year-old lady with a suspicious right upper lung mass and with a significant smoking history. Intense FDG uptake corresponding to the mass (arrows) indicates a malignant etiology, unless proved otherwise; however, a resection biopsy showed only inflammation and necrosis

Several studies have shown FDG-PET to be highly accurate in differentiating benign from malignant pulmonary lesions, with a sensitivity of greater than 95% and a specificity of 75-80%.[36] The sensitivity is affected by the lesion size (lower sensitivity for smaller lesions), its location (lower sensitivity for lesions located near the thoraco-abdominal interface because of respiratory motion or for those close to a ‘hot’ myocardium) and sometimes patient's body habitus (lower sensitivity due to high noise in large/ heavy patients). The high negative predictive value of FDG-PET scan makes it a very useful modality, except in sub-centimeter nodules, for reasons described above. Pure BAC and very low grade or very well-differentiated adenocarcinomas may demonstrate low FDG activity as well. Therefore, follow-up with CT scans is recommended for suspicious lung lesions to ascertain their stability. The specificity varies depending on the population being studied and is lower in areas where sarcoidosis, fungal granulomas (like histoplasmosis or coccidioidomycosis) and tuberculosis are more prevalent [Figure 15]. False positive lesions may also be seen with active infectious / inflammatory lesions like necrotizing granulomas [Figure 16].

Certain PET imaging centers practice dual-time point imaging (at 1 and 1.5-2 h) to improve the specificity for malignant pulmonary lesions.[37–39] The hypothesis of dual-point imaging is based on studies that show increased FDG accumulation in malignant lesions 60 to 90 min after injection, whereas uptake in infectious /inflammatory lesions tends to decrease with time. Early studies showed that dual-time point imaging improves the accuracy of FDG-PET, but considerable overlap between malignant and infectious/ inflammatory lesions is still seen.

**Figure 17:**
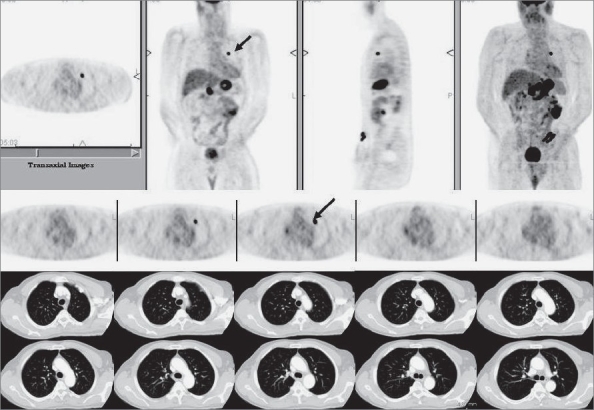
PET and CT scan images of a 64-year-old man with a descending/ sigmoid colon mass on recent colonoscopy. The PET scan reveals an intensely FDG-avid descending/ sigmoid colon malignant lesion with relatively widespread metastatic disease in the abdomen and a single intense left upper lobe metastatic lesion (arrows in the axial and MIP images in the top row and in axial images through the upper chest in the middle row). However, a contrast-enhanced chest CT scan done the next day reveals no corresponding lesion (bottom two rows), though there was evidence of pulmonary embolism

An important caveat relates to the incidental detection of a focal intense lesion in the lung on FDG-PET imaging without a corresponding CT correlate [Figure 17]. Since the newer CT scanners are very sensitive for the detection of small (<5 mm) lung nodules, in the absence of a true CT lesion, this is usually an artifact and may be due to a faulty injection technique. Such artifacts are typically seen if blood is withdrawn into the injection syringe before injecting FDG (to ensure correct intravenous access). Sometimes, misregistration between the PET and CT images due to respiratory motion (especially at the lung bases / liver dome) may also suggest a lesion on FDG-PET imaging, though a review of the CT scan images will immediately resolve the issue.

**Figure 18:**
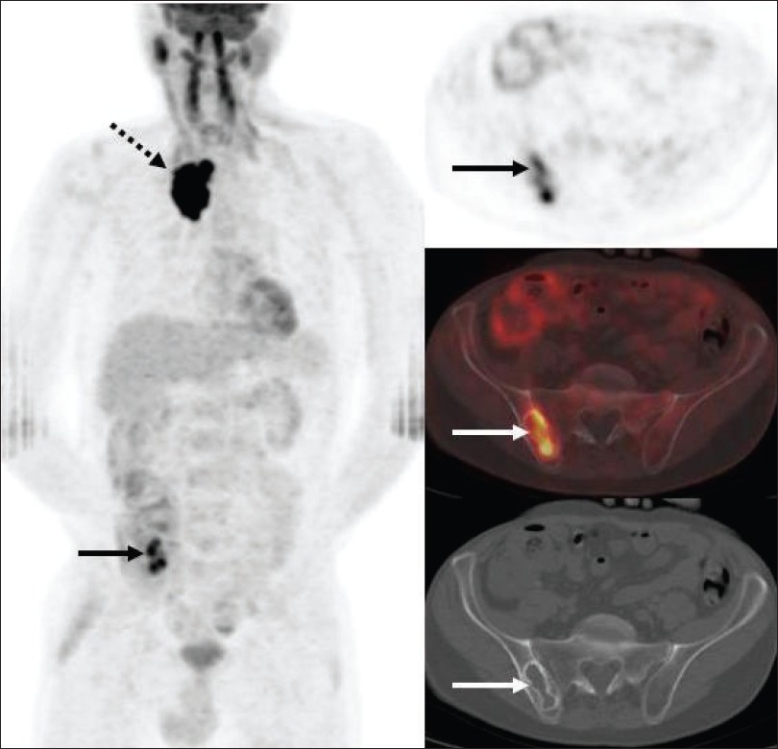
Axial PET (top right), fused PET/CT (middle right) and CT (bottom right) images at the level of the pelvic inlet, with an MIP image (on left), from a PET study of a 71-year-old man with a right upper lobe cancer. In addition to showing intense uptake in the right upper lobe lung cancer (dotted arrow), the study also shows an unsuspected right iliac bone metastasis, upgrading the patient to stage IV disease

FDG-PET imaging is an important component of the TNM staging workup of NSCLC. Anatomical imaging modalities such as CT scan or MRI are superior to FDG-PET and PET/CT (using no intravenous or oral contrast and a low-dose CT scan for attenuation correction and anatomic localization only) for evaluation of the ‘T’ stage. However, FDG-PET imaging is superior to CT imaging for ‘N’ and ‘M’ staging. FDG-PET has proven to be more accurate in assessing nodal involvement and it can accurately identify metastatic disease in normal-sized nodes on CT. It can also better differentiate lymph node metastases from reactive, but enlarged nodes as seen on CT. The sensitivity and specificity of FDG-PET imaging in N3 disease (contralateral mediastinal and ipsilateral or contralateral supraclavicular nodal involvement) is greater than 90% and its overall accuracy for assessing mediastinal nodal involvement is around 85%. However, caution is recommended in N0 disease per PET since micrometastases cannot be detected by FDG-PET imaging and invasive staging of intra-thoracic lymph nodes may yield positive nodal disease.[40] The specificity is again affected by infectious/ inflammatory conditions like sarcoidosis, histoplasmosis and tuberculosis. FDG-PET imaging is also superior to other imaging modalities for detecting distant metastatic disease outside the brain. An unexpected distant metastatic lesion may be seen in up to 12% of patients[41] [Figure 18].

**Figure 19:**
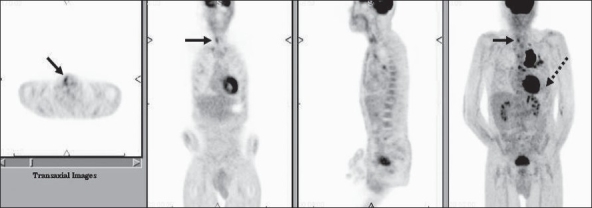
PET study of a 58-year-old man with left lung cancer - with axial, coronal, sagittal and MIP images from left to right focusing on the vocal cord region. The PET scan shows a left lung cancer with mediastinal nodal metastases. It also indicates left recurrent laryngeal and phrenic nerve impairment as evidenced by lack of FDG activity in the left vocal cord (arrow pointing towards physiologic uptake in the right vocal cord) and elevation of the left hemidiaphragm (dotted arrow)

FDG-PET imaging can also detect functional impairment of other structures due to lung carcinoma. Bulky metastatic mediastinal adenopathy can frequently compress/ involve the recurrent laryngeal and phrenic nerves. Elevation of the ipsilateral hemi-diaphragm or lack of FDG activity in the ipsilateral vocal cord with physiologic or compensatory increased FDG activity in the contralateral vocal cord indicates ipsilateral phrenic nerve or recurrent laryngeal nerve impairment respectively [Figure 19].

**Figure 20:**
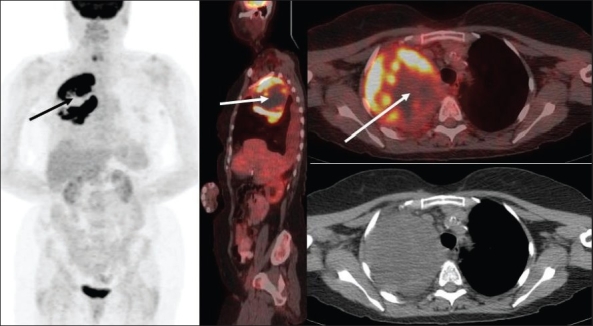
Axial fused PET/CT (top right) and CT (bottom right) images at the level of the upper lungs, with a sagittal fused PET/CT image (middle) and an MIP image (on left), from a PET study of a 71-year-old lady with a large right upper lobe mass. Bronchoscopic evaluation and two CT-guided biopsies failed to reveal malignancy. PET scan clearly shows a large malignant right upper lung mass with a large ‘cold’ central necrotic area (arrows) and intense FDG uptake peripherally. A third CT-guided biopsy from the periphery of the mass based on the PET findings confirmed NSCLC

FDG-PET imaging is presently considered the most accurate noninvasive means of staging a biopsy-proven NSCLC (excluding brain metastases). In addition to its high accuracy, FDG-PET imaging can also help make the diagnostic and staging workup of NSCLC more efficient and cost-effective. FDG-PET imaging can help to guide diagnostic biopsies and improve the yield from tissue sampling. We frequently see lung masses with intense FDG uptake but with a bronchoscopic workup negative for malignancy. These lesions are subsequently proven to be malignant on additional biopsies in a majority of such cases. Even CT-guided biopsies of lung masses may be falsely negative if a necrotic area is targeted during biopsy. Figure 20 shows a large right lung mass that had a bronchoscopic workup, as well as two CT-guided biopsies that were all negative for malignancy. The PET scan clearly showed a large area of central necrosis to be responsible for the negative biopsies and a third CT-guided biopsy from the peripheral portion of the lesion that was intensely FDG avid, finally confirmed a diagnosis of NSCLC. Also, if a distant metastatic lesion is identified on an FDG-PET scan for a lung mass, a biopsy from that site alone can prove a stage IV lung neoplasm, sometimes obviating the need for biopsy of the primary lesion (especially when lung biopsy may be deemed risky, as in patients with severe COPD). In general, a biopsy of the site suggesting the highest stage of the disease as identified on FDG-PET imaging is recommended.

FDG avidity of the lung lesion also provides prognostic information. Since FDG uptake correlates with tumor growth rate, doubling time and proliferation, intensely FDG-avid lesions tend to be more aggressive with poorer prognosis. SUV is considered an independent prognostic factor and tumors with SUV >10 have a median survival of less than a year.[42]

In patients undergoing curative, palliative or neoadjuvant radiation therapy, FDG-PET also helps in radiation therapy planning. Incorporation of the PET data in the process of determining the radiation therapy ports allows more accurate tumor localization, leading to tighter contouring and smaller biological volume calculation and sparing of uninvolved lung parenchyma.[43] This may potentially translate into better-tolerated radiation therapy with less pulmonary side effects and also better control of the disease by avoiding treatment misses to involved areas, as well as allowing the maximum possible radiation dose to the smallest possible target volume. In addition, PET/CT with respiratory gating (also referred to as ‘4D PET/CT’) may allow even better targeting of the tumor by taking into consideration respiratory motion of the lesion in the therapy planning protocol.[44]

**Figure 21 (A, B):**
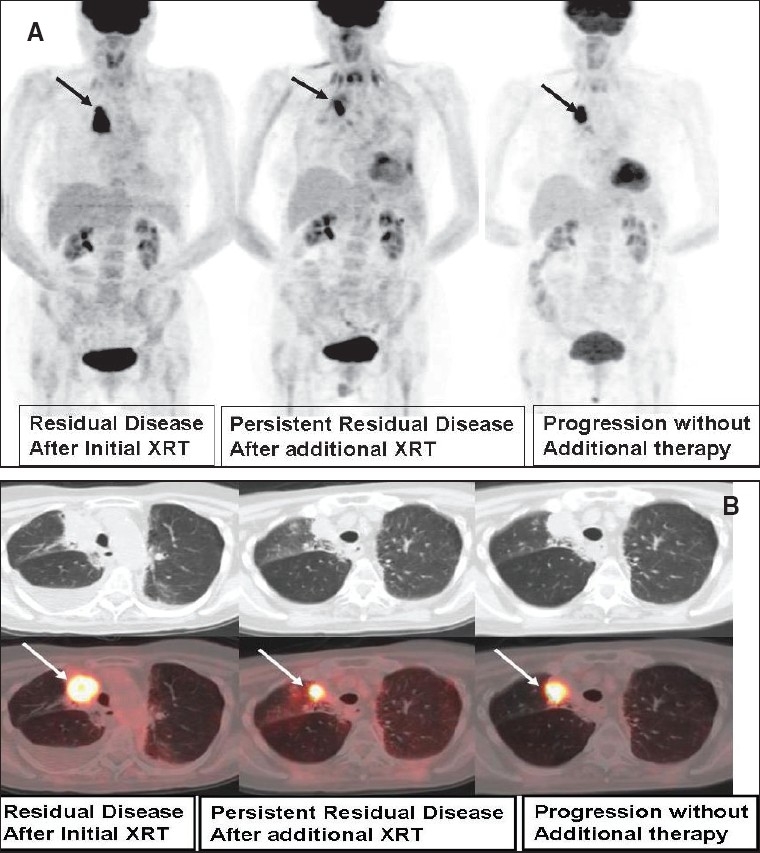
PET studies of a 79-year-old lady with a remote history of right lung cancer, with MIP (A) and axial (B) CT scan (top row) and fused PET/CT (bottom row) images at the level of the right upper lobe mass from three consecutive PET scans. Initial study after radiotherapy (left) shows an intensely FDG-avid right upper lung mass, proven to be recurrent neoplasm. A repeat PET study (middle) 4 months after completing concurrent chemoradiation therapy reveals incomplete response with small-to-moderate amount of intense residual disease. Since post-radiation therapy changes can last and remain intense for a considerable interval of time, the patient was observed without additional therapy. A follow-up PET study (right) 3 months later (7 months after completing chemoradiation) reveals disease progression

**Figure 22 (A, B):**
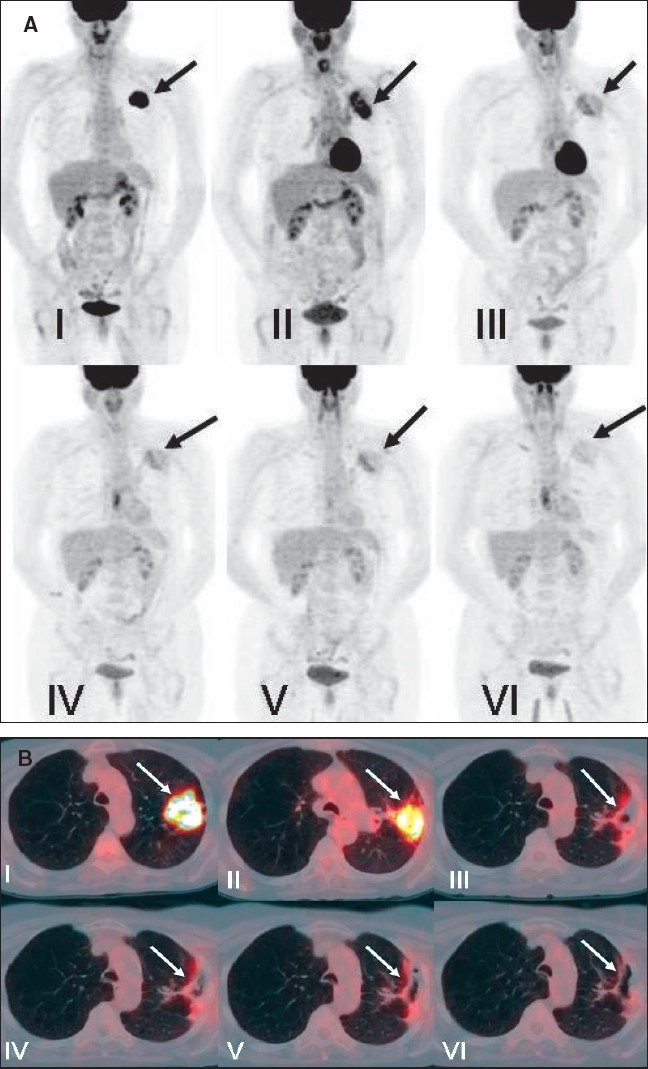
PET studies of a 58-year-old lady with left lung cancer. MIP images (A) and axial fused PET/CT images (B) of the left upper lobe mass of six consecutive PET scans are shown. The initial study (I) shows intense FDG uptake in the left upper lung neoplasm without loco-regional nodal or distant metastatic disease. A repeat study (II) 5 months after completing radiation therapy shows interval improvement with significant residual uptake. Subsequent studies (III-VI) 7 months, 11 months, 15 months and 22 months after completing therapy reveal gradual improvement in the intensity of uptake without evidence of residual/ recurrent disease

FDG-PET imaging is also playing an increasing role in restaging lung cancer patients to assess response to therapy (especially after radiation therapy) and to detect recurrence. It is superior to CT scan for differentiating post-therapy changes (after surgery, chemotherapy and/ or radiation therapy) from residual or recurrent malignant disease [Figure 21] and the additional information from the CT images imparts greater specificity to the interpretation of the PET findings. The timing of assessment after radiation therapy is crucial; and frequently, if performed earlier, there is high uptake in the radiation therapy port. This may persist for a considerable amount of time (3-6 months) and may be quite intense in the presence of frank radiation pneumonitis. Although the intensity of FDG activity is expected to subside in 6 months or so, it may remain elevated for even longer [Figure 22] and generally stable findings are considered negative for residual or recurrent malignant disease. Increasing intensity, focal intense uptake or a new intense lesion in or around the radiation treatment area are suspicious for recurrence. At our center, we are currently evaluating the changes in FDG-PET findings over a prolonged period of time after localized radiosurgery using tomotherapy for lung cancer. An 80% decrease or more in SUV predicts a high likelihood of complete response irrespective of the subtype of NSCLC, type of neoadjuvant therapy and final absolute SUV of the lesion.[45]

SCLC also shows intense FDG uptake. However, FDG-PET imaging is currently not approved by CMS for the workup of SCLC because it is frequently metastatic at the time of diagnosis and the addition of FDG-PET imaging is not felt to have a clinical impact on patient management and outcome. SCLC is generally staged as limited- and extensive-stage disease; and potentially, a small number of limited-stage SCLC patients (with T1-T2, N0, M0 disease) may be curable with aggressive surgical resection followed by chemotherapy. The major consideration in staging SCLC is to determine the need to add radiation with chemotherapy since limited-stage patients are considered for combined chemoradiation therapy with curative intent. FDG-PET has a potential role in SCLC to confirm limited-stage disease or upstage these patients to extensive-stage disease. However, this is all still under investigation.

**Figure 23:**
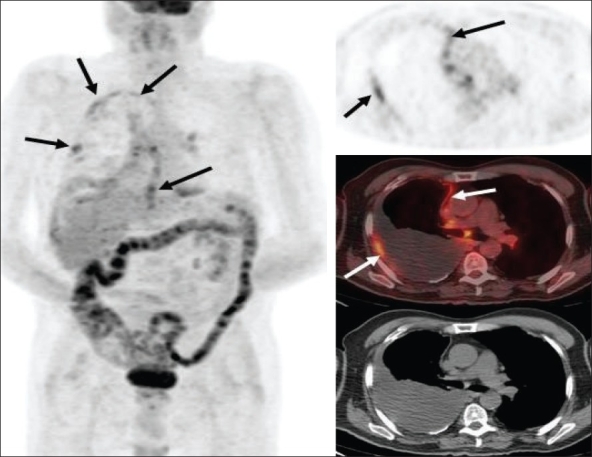
Axial PET (top right), fused PET/CT (middle right) and CT scan (bottom right) images at the subcarinal level, with an MIP image (on left), from a PET study of a 77-year-old man with a large right pleural effusion, right lower lung atelectasis and mediastinal adenopathy. Cytology from the pleural fluid was positive for malignant cells. The study shows a pleura-based malignant process in view of the mildly increased FDG activity along the right lung pleura with scattered, more intense pleura-based nodularities (arrows) and metastatic mediastinal adenopathy. Pleural biopsy confirmed mesothelioma

**Figure 24:**
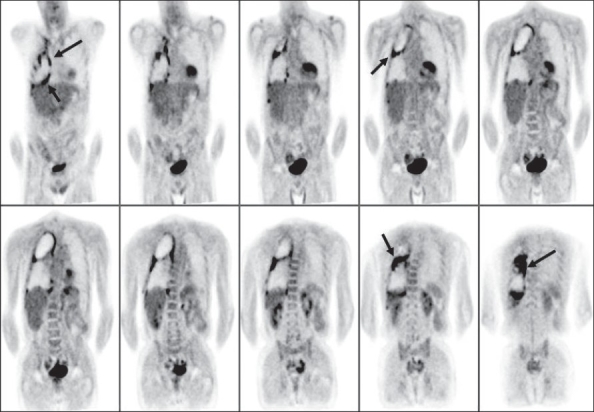
Thirteen millimeter coronal PET images in a 64-year-old man with an abnormal CT scan of the chest. The patient had a history of talc pleurodesis for recurrent right pleural effusion, which is responsible for the intense pleura-based FDG activity around the right lung

FDG-PET imaging is also useful in evaluating pleural malignancies like mesothelioma [Figure 23], which tend to show intense FDG uptake. In equivocal cases, pleural effusions may also be suggestive of malignancy if they show even mild increased FDG activity. Focally intense abnormalities within the pleural effusion/abnormality may help guide biopsy. In the evaluation of lung and pleural malignancies with FDG-PET imaging, it is important to bear in mind that talc pleurodesis shows intense FDG uptake [Figure 24] that is usually sustained for a prolonged period of time and can sometimes wax and wane over time. Obtaining a relevant clinical history in such cases is obviously of paramount importance for avoiding mistakes in interpretation.

### Lymphomas

Lymphoma is the fifth most common cancer in the US and is one of the few malignancies whose incidence is rising.[4] Lymphoma is a heterogeneous group of neoplasms involving the immunological system and has several classifications. Broadly, it is classified into two major types: Hodgkin's disease (HD) and non-Hodgkin's lymphoma (NHL), both of which have several subtypes. CT scan is considered the predominant modality of choice for lymphoma workup, although it is likely to be replaced by FDG-PET/CT imaging in the near future. Traditionally, gallium (Ga-67) scintigraphy has been used for staging and restaging lymphoma. However, FDG-PET offers several advantages over Ga-67 imaging in the form of better dosimetry (lower radiation dose), quicker scans (1.5-2 h *vs.* 2-3 days or more), higher sensitivity (due to superior spatial resolution, lesser physiologic bowel uptake and better contrast resolution), better quantification potential (and hence better prognostic information) and superior assessment of response to therapy.[46–51] FDG-PET imaging has an important role in all aspects of lymphoma workup: initial diagnosis, staging, grading, restaging (evaluation for recurrence or residual disease), assessing response to therapy and surveillance (especially for higher-stage disease initially). Lymphoma is perhaps the most frequent oncologic condition for which FDG-PET imaging is performed.

**Figure 25:**
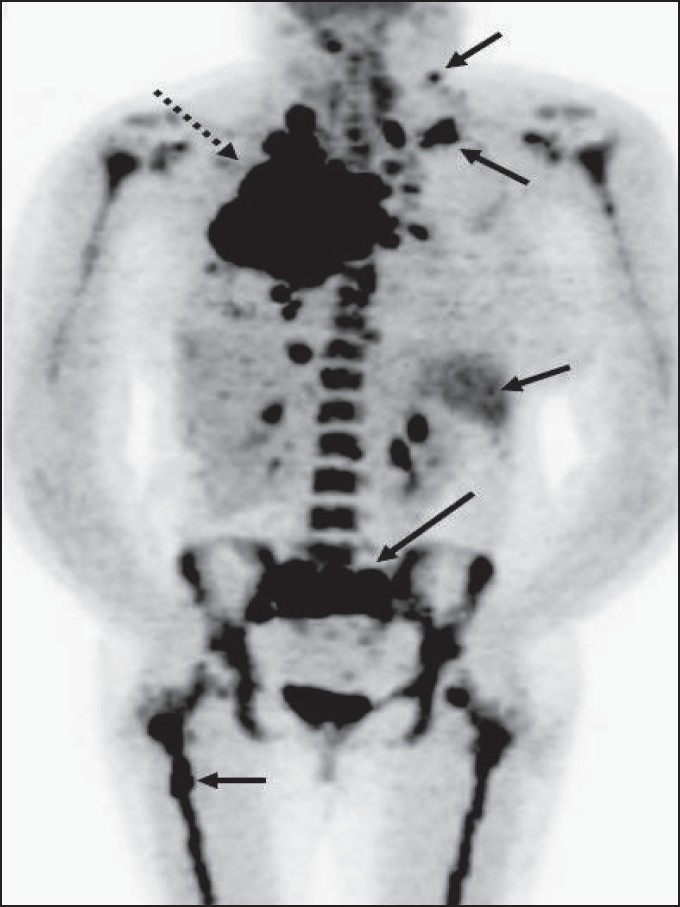
MIP image from a PET study of a 35-year-old lady presenting with a large FDG-avid right lung mass (dotted arrow). The widespread neoplastic disease, along with increased splenic and bone marrow uptake (arrows), suggests lymphoma as the possible underlying etiology. The patient was proven to have HD

**Figure 26:**
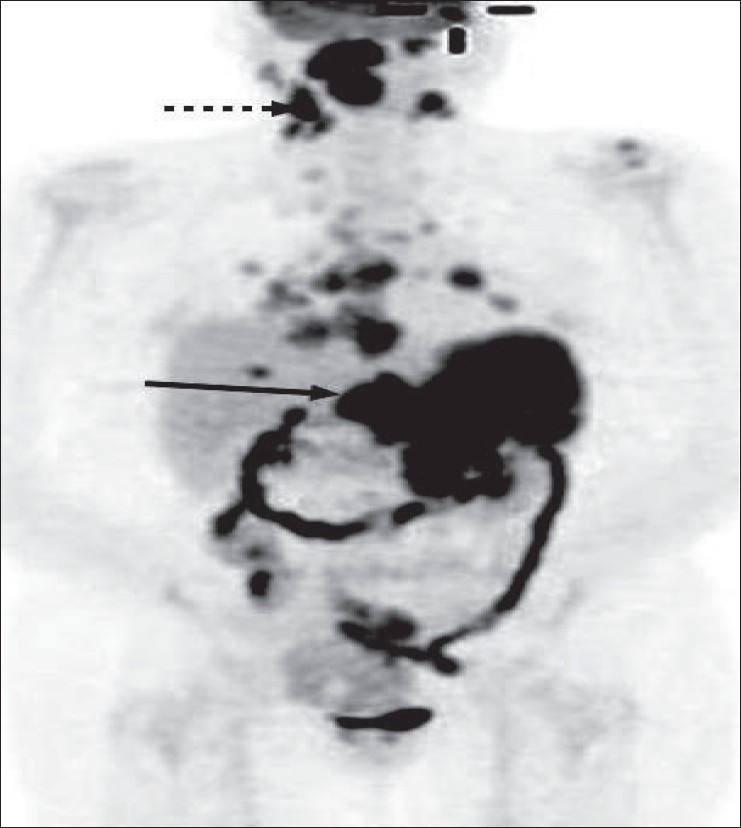
A 63-year-old lady presenting with abdominal pain was found to have a retroperitoneal mass. Biopsy revealed follicular lymphoma. MIP image from a staging PET study shows widespread involvement, the retroperitoneal mass showing intense FDG uptake (arrow). However, the neck nodes also show intense uptake (dotted arrow); and if this was known earlier, it would have presented a more accessible option for biopsy and confirmation of the diagnosis

Tissue diagnosis is the norm in lymphoma workup. Although FDG-PET does not image lymphoma cells *perse* (or any other cancer cells, for that matter) but rather images the intracellular accumulation of FDG-6-phosphate (intracellular glucose, for all practical purposes), the pattern of FDG-avid lesions may suggest a diagnosis of lymphoma when the scan is being performed for some other reason [Figure 25]. In addition, when a diagnosis of lymphoma is suspected, it may suggest another more accessible site for tissue diagnosis [Figure 26]. It is important to note that almost all lymphoma types show increased FDG uptake,[52,53] including low-grade NHL (unlike Ga imaging). Though MALT lymphomas have been traditionally considered not FDG avid,[54] a significant number (about 60%) have now been reported to show increased uptake.[52,53]

**Figure 27:**
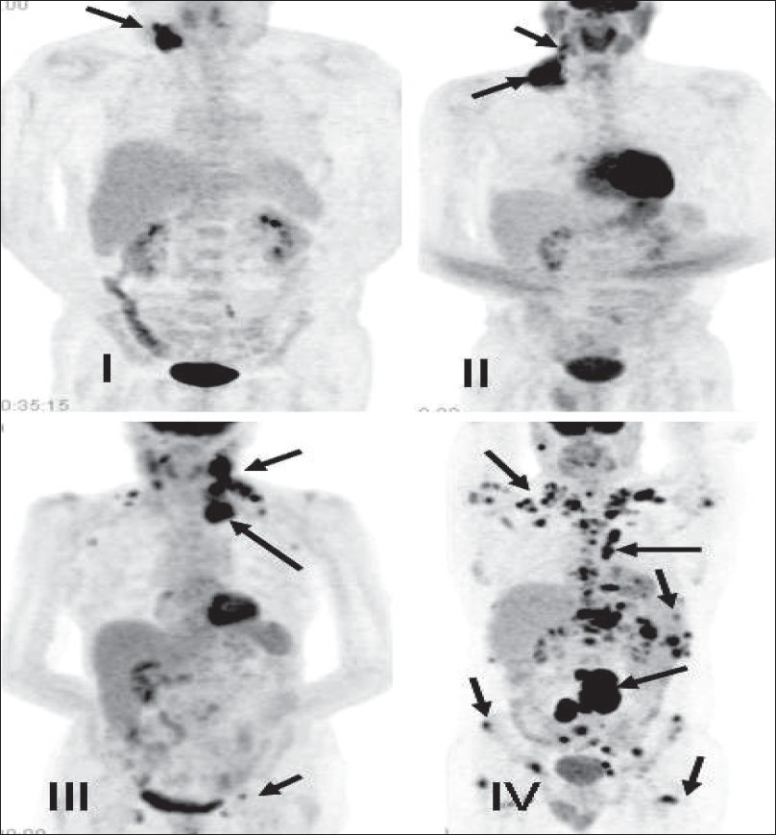
MIP images from PET studies of four different patients with lymphoma, showing stage I (upper left), stage II (upper right), stage III (lower left) and stage IV (lower right) disease. Arrows indicate various lesions that define the disease stage

The American Joint Committee on Cancer (AJCC) has based the staging of lymphoma on the Ann Arbor classification that considers the number of nodal regions involved, the extent of involvement and extranodal involvement along with modifiers according to the presence or absence of ‘B’ symptoms. FDG-PET imaging is crucial for staging lymphomas [Figure 27] and is considered either as good as or better than CT scanning. It is superior for extranodal disease detection, especially splenic involvement.[55–57] It is complementary to bone marrow biopsy for assessing bone marrow involvement.[58,59] Although it can miss low-grade, diffuse involvement and may have limited specificity for diffuse involvement versus hematopoietic stimulation from other reasons, it can detect focal bone marrow involvement that may be missed on bone marrow biopsy and can also help provide a better site for bone marrow biopsy. It generally upstages about 20% of patients (although a wide range is reported in the literature) and affects management in a significant number of patients.[59–62] FDG-PET/CT is considered better than CT alone or FDG-P*ET al*one for staging lymphoma and has been shown to be cost-effective and accurate when the initial scans have guided further conventional imaging.[63]

**Figure 28:**
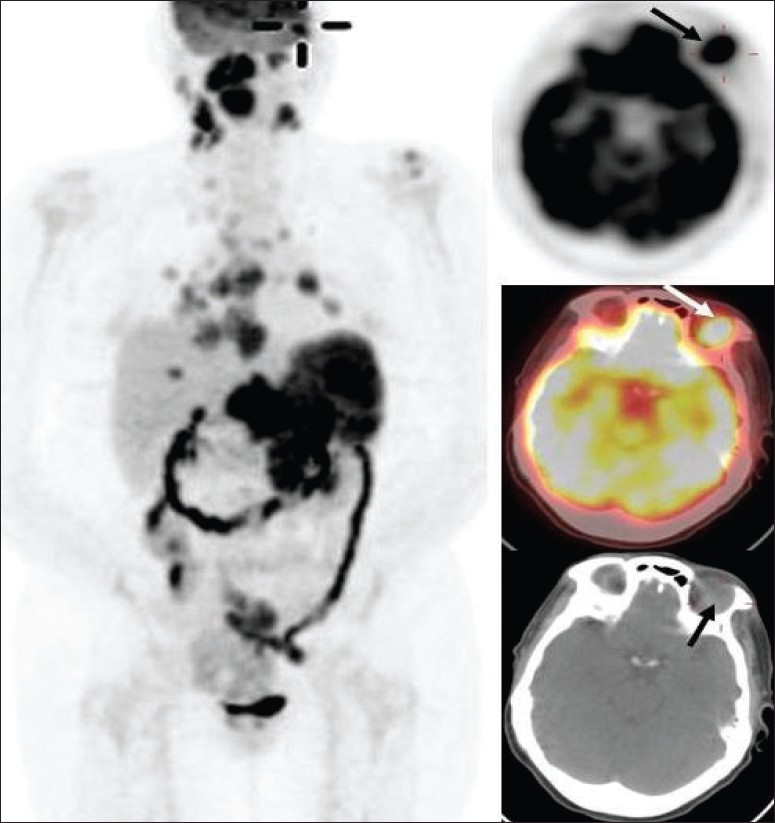
:Axial PET (top right), fused PET/CT (middle right) and CT scan (bottom right) images at the level of the frontal sinus, with an MIP image (on left), from the same patient as in Figure 26. The arrow points towards ocular involvement, although the patient did not have any ocular complaints at the time

**Figure 29:**
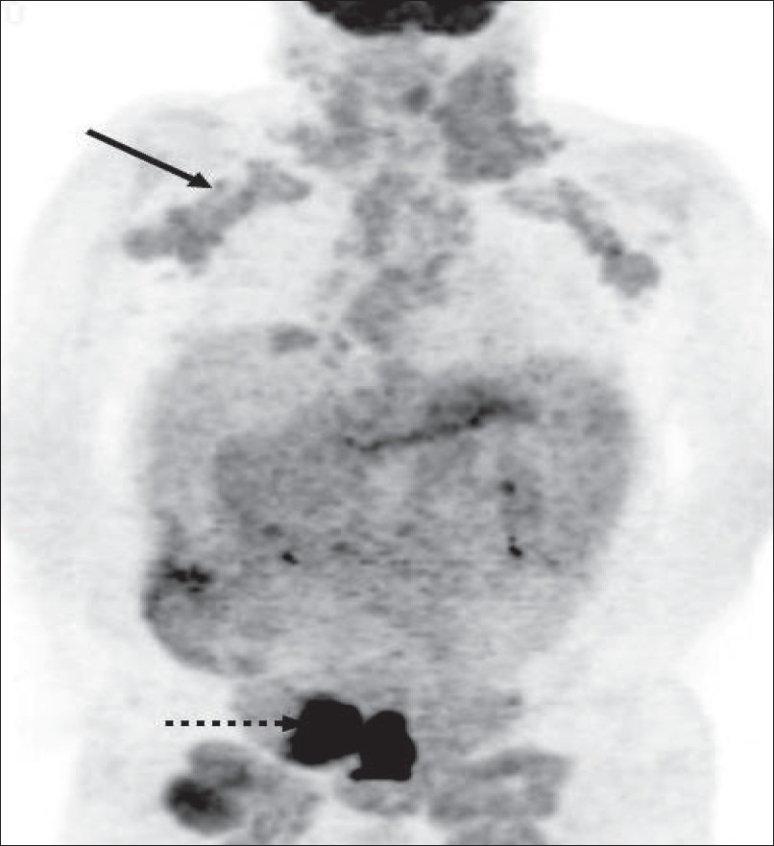
MIP image from a PET study of a 69-year-old man with NHL. Biopsy from the enlarged right axillary nodes (arrow) showed a low-grade lymphoma. The PET study shows moderate uptake in the bilateral neck; supraclavicular, axillary, mediastinal and inguinofemoral lymphadenopathy consistent with the biopsy-proven low-grade lymphoma. However, there is an additional concurrent intense focus in the right pelvis/ iliac region (dotted arrow), indicating a high-grade neoplastic lesion in the same patient

The intensity of FDG activity generally correlates with the grade of lymphoma on histology and provides prognostic information that may impact management decisions.[64] Aggressive lymphomas usually show an SUV ≥13 in the most intense lesion and indolent lymphomas usually show an SUV of ≤6 in the most intense lesion; though there is significant overlap.[65] Not infrequently, there is incongruence between the histologic grade reported and the intensity of FDG activity and usually FDG-PET imaging correctly predicts the nature of the lesion (especially if the FDG-PET shows an intense lesion suggesting high-grade/ aggressive disease and the histology shows low-grade lymphoma). The patient depicted in Figure 26 had a diagnosis of low-grade lymphoma on histology, but the PET study clearly and correctly showed the aggressive nature of the disease based on the intensity as well as the widespread disease, also involving the left orbital cavity [Figure 28]. Lesions of two different grades are known to coexist in the same patient, as well [Figure 29].

**Figure 30 (A, B):**
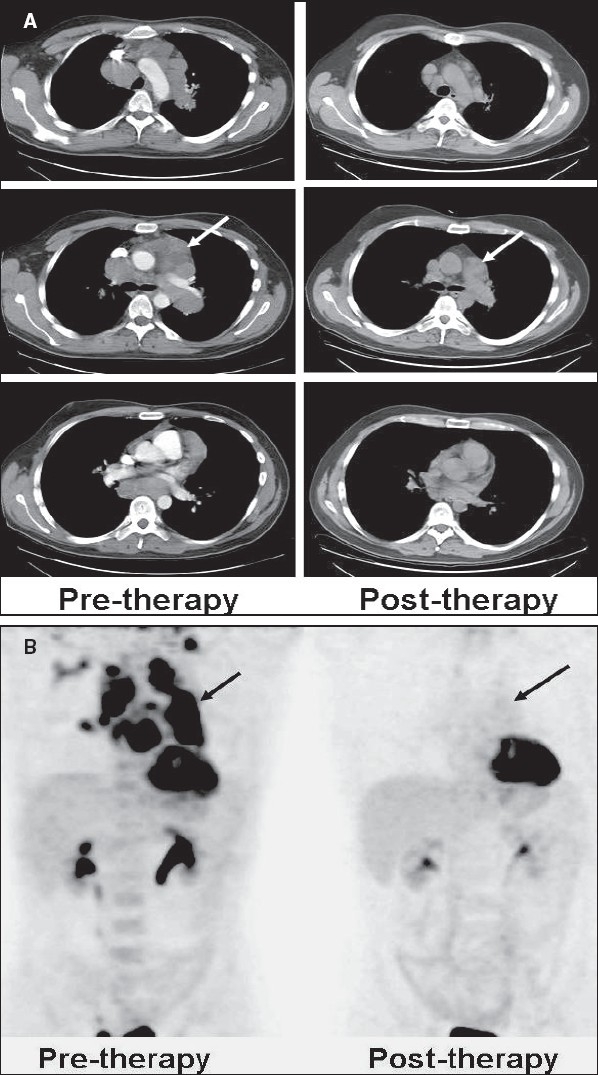
Pre-therapy (left) and post-therapy (right) axial CT scan images (A) of a patient with lymphoma indicate incomplete response to therapy with residual lymphadenopathy in the mediastinum (arrows). The pre-therapy (left) and post-therapy (right) MIP images (B) from a PET study of the same patient (performed within a week of the respective CT scans) show complete remission (arrows)

Ga-67 scintigraphy and CT scan have been the mainstay for assessing response to therapy in lymphoma patients; and the Chesson classification, based mainly on size criteria for scanning, has been in vogue since 1999.[66] Based on their response to therapy, this system classifies patients into one of several groups: Complete Response (CR), Unconfirmed CR (CRu), Partial Response (PR), Stable Disease (SD) and Progressive Disease (PD). With the advent of FDG-PET, these criteria have been shown to be clearly suboptimal [Figure 30] and were recently revised - with more reliance on FDG-PET findings.[67] The revisions have increased the number of CRs, eliminated the category of CRu and helped discern differences in progression-free survival (PFS) between CR and PR patients.

**Figure 31:**
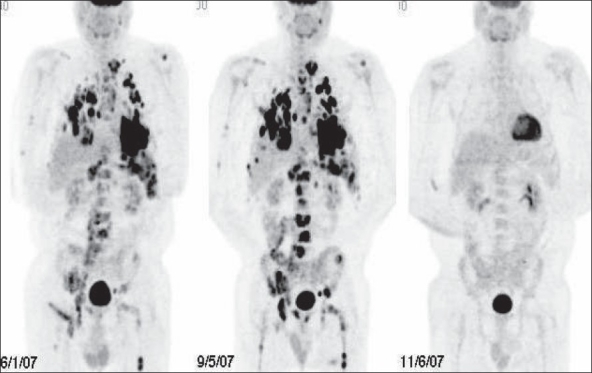
MIP images from PET studies of the same patient with lymphoma, shown in Figure 30. Pre-treatment staging PET study (left image) shows widespread involvement. A repeat PET study after two courses of chemotherapy (middle image) shows disease progression. Another PET study after two cycles of changed chemotherapy shows excellent response - to near-complete remission

FDG-PET imaging can correctly assess response to therapy as early as 7 days after the first chemotherapy cycle. Recent oncology practice management guidelines recommend an FDG-PET scan after two cycles of chemotherapy (around 3 weeks after the last treatment or just before the next cycle) to assess response and to then proceed with further therapy based on the results of this scan[68,69] [Figure 31]. It is important to be aware of the known pitfalls and lesions causing false positives while interpreting these studies (like increased uptake in the thymus due to rebound thymic hypertrophy or inflammatory uptake and increased uptake in the bone marrow due to cytokine stimulation). Assessing response after radiation therapy is more complex than monitoring chemotherapy. The timing of the post-treatment FDG-PET scan is of paramount importance for assessing accurate response and there is still no consensus as regards the optimal timing for FDG-PET imaging after radiation therapy.[67] Studies and observations from other neoplasms indicate that FDG-PET imaging may be positive due to radiation-induced inflammation and this may last for a significant amount of time (many months). At our center, we recommend an interval of at least 3 months after completing radiation therapy, before advising a repeat scan. In such cases, the pattern of uptake (within the radiation therapy port, geometric configuration), the pre-therapy scan and the clinical information help to improve specificity.

FDG-PET imaging cannot assess microscopic disease and remission (generally meaning less than 1 billion tumor cells) is not equal to tumor eradication or cure.[70] However, a negative FDG-PET scan at the end of therapy does indicate excellent prognosis with sustained progression-free survival. Conversely, persistent increased FDG uptake during or at the end of therapy indicates a high risk of relapse.[71,72] Hence the current trend is towards assessing response to therapy with FDG-PET imaging early during chemotherapy and then again at the end of therapy.

FDG-PET also has proven value in assessing disease recurrence and residual disease after therapy.[73] A mass may never shrink completely but may be completely inactive based on FDG-PET imaging. Also, a mass may remain considerably large, but only a portion of the mass may be active on FDG-PET. In such cases, FDG-PET findings have been proven to be more accurate than CT scan for predicting residual disease. It should be noted that large masses that were intensely FDG avid prior to therapy might retain faint activity for a long period of time, usually a result of post-therapy inflammatory changes. In addition, FDG-PET also detects disease recurrence earlier; and in patients presenting initially with higher-stage disease (stage III or IV), surveillance with FDG-PET imaging may be cost-effective for detecting recurrences.[74,75]

**Figure 32:**
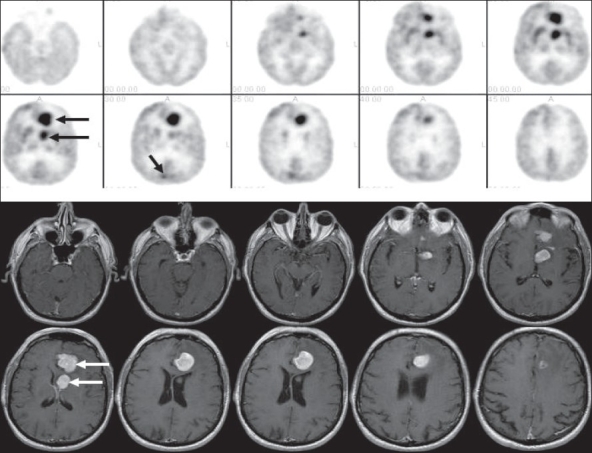
Axial brain images with FDG-PET (top two rows) and MRI (bottom two rows) in a patient with CNS lymphoma. Although it is difficult to assess malignant lesions in the brain, CNS lymphoma lesions are usually very intense on FDG-PET imaging; and as shown in this example, sometimes more lesions can be seen with FDG-PET brain imaging as compared to brain MRI (arrows)

**Figure 33:**
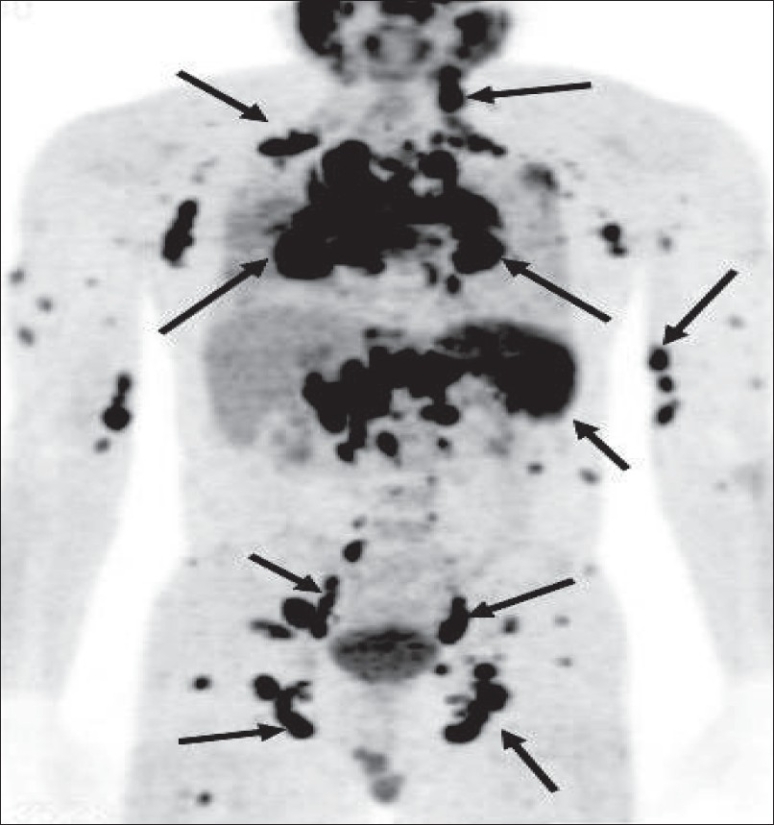
MIP image from a PET study of a 42-year-old man with widespread intensely FDG-avid lesions involving lymph nodes in the neck, supraclavicular regions, axillae, chest (bilateral hilar and mediastinum), abdomen (para-aortic, retroperitoneal and iliac chain) and inguino-femoral regions as well as the spleen along with multiple subdermal/ subcutaneous lesions (arrows), mimicking lymphoma. Biopsy showed sarcoidosis in the nodes, as well as in the subdermal/ subcutaneous lesions

Thus, FDG-PET imaging plays a crucial role in the workup and management of lymphoma. However, it does have certain limitations.[76] Smaller lesions (<7-8 mm) and certain types of lymphoma lesions (very low grade lymphoma, few MALT and mantle cell lymphomas) may be missed. Certain lesions may be masked (renal lesions due to physiologic excretion of FDG by kidneys, GI tract lesions due to intense GI tract uptake in a few patients, patients with intense brown fat uptake, altered biodistribution of FDG due to hyperinsulinemia or rigorous physical activity prior to the FDG-PET imaging). FDG-PET imaging generally has limited sensitivity for detecting metastatic brain lesions. However, usually cerebral lymphoma lesions are ‘hot’ [Figure 32]; and in HIV patients with brain lesions, FDG-PET imaging of the brain has become the test of choice to differentiate between toxoplasmosis (not ‘hot’ on FDG-PET) and lymphoma.[77–79] Some other conditions may mimic lymphoma, sarcoidosis being a frequent culprit in our part of the US [Figure 33]. Nodal uptake due to radiotracer administration infiltration artifact and intense focal GI tract lesions (like benign lymphoid hyperplasia, inflammatory or adenomatous polyps) may also cause problems with interpretation.

### Melanoma

Melanoma is the most serious of skin cancers and is much more frequent in white-skinned patients. In 2007, an estimated 60,000 new cases are expected in the US, with about 8,000 deaths.[4] Diagnosis requires a biopsy and thorough histopathological examination to assess lesion thickness (Breslow's Index) and depth of invasion (Clark's levels), which are important for staging. Early-stage diseases (stages I and II as per AJCC) are potentially curable, with high 5-year survival rates, but the mortality rate is quite high for advanced disease (especially stage IV).

Melanoma is considered one of the most FDG-avid tumors.[80] Imaging with FDG-PET is considered more accurate than standard diagnostic clinical procedures, including clinical examination, radiography, CT scan, MRI, ultrasound and hepatic enzymes.[81,82] However, FDG-PET imaging has a very limited role in early-stage disease. The ‘sentinel node’ technique is an established modality with high sensitivity, for assessing regional lymph nodes in such cases; and FDG-PET imaging is not an alternative due to its limited sensitivity in detecting micrometastases and low-volume disease (<78 mm^3^).[83] Moreover, the incidence of regional or distant metastatic disease in early-stage asymptomatic patients after adequate treatment of the primary lesion (wide local excision with sentinel node biopsy and nodal basin dissection if necessary) is low enough that routine evaluation with FDG-PET imaging in such patients is not justified. However, in patients with advanced disease, FDG-PET imaging may serve as an important study for overall assessment of the disease (excluding brain and small pulmonary lesions). Although in a patient with confirmed widespread metastatic melanoma, additional information about extent of disease by FDG-PET imaging may not have significant impact on patient management and outcome; it does help in certain cases. When patients are being considered for surgical resection of isolated distant metastatic lesions, FDG-PET imaging is used to accurately assess disease extent. Additional lesions identified on FDG-PET imaging make surgical resection unwarranted. FDG-PET may have a clinical impact on management in 53% of patients by either upstaging (more frequently) or downstaging patients.[84] Frequently, unexpected nodal or distant metastases and cutaneous/ subcutaneous lesions are detected on FDG-PET imaging. Sometimes, lesions felt to be definite visceral metastases on other conventional modalities (like CT or MRI) may be shown to be benign on PET, thus downstaging these patients and making them candidates for potentially curable treatments. However, caution is recommended with pulmonary and intracranial lesions due to reasons described in earlier sections.

**Figure 34:**
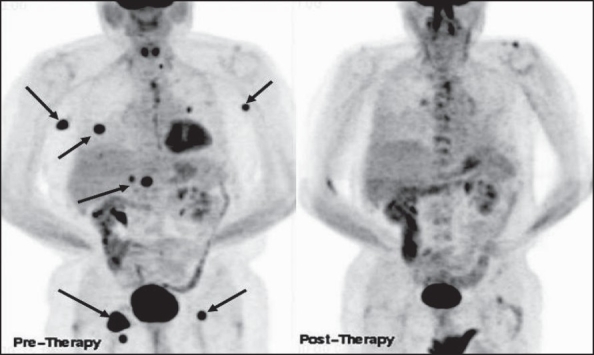
MIP images from pre-therapy (left image) and posttherapy (right image) PET studies of a 66-year-old man with metastatic melanoma (arrows), showing excellent response to therapy with complete remission

**Figure 35 (A, B):**
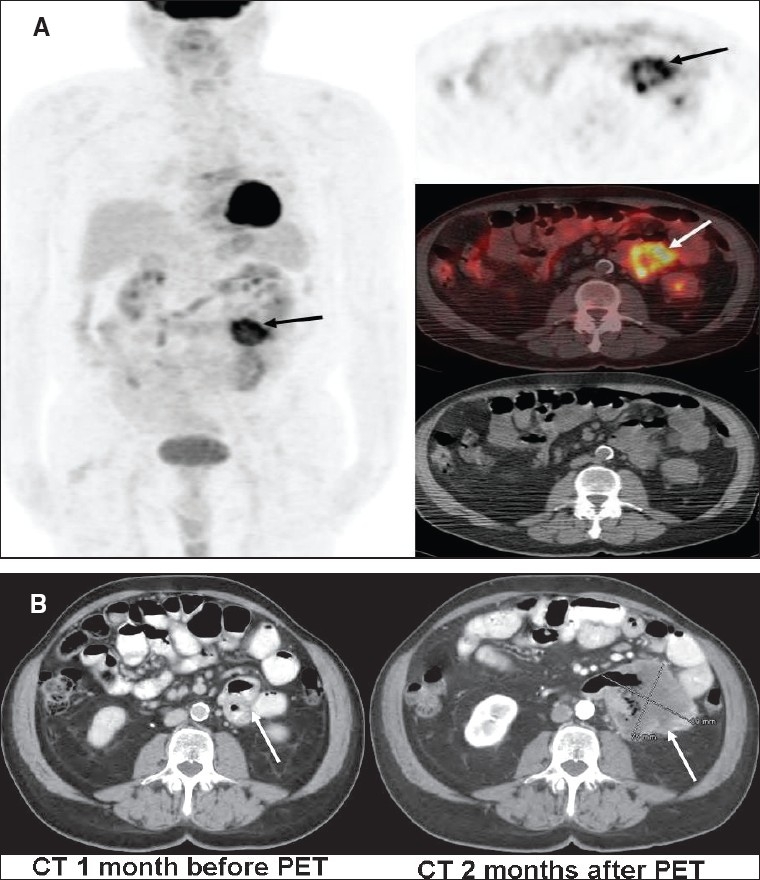
Axial PET (top right), fused PET/CT (middle right) and CT scan (bottom right) images at the level of the mid-abdomen, with an MIP image (on left), from a PET study (A) of a patient with treated melanoma. The images show an intense gut-associated focus in the left abdomen (arrows), strongly suspicious of recurrence. However, a structural lesion that could be biopsied was evident only 2 months later (B) on a CT scan (right image). No structural lesion was evident even retrospectively on a prior CT scan (left image)

FDG-PET has a more established role in assessing response to therapy (especially with the newer treatment options) [Figure 34] and in detecting recurrent disease [Figure 35].[85] Thus, FDG-PET may provide a cost-effective tool in managing high-risk patients of malignant melanoma for initial staging and management planning, assessing response, detecting recurrence and guiding additional workup. The recent mainstream use of combined PET/CT imaging may further improve the sensitivity and specificity of this imaging modality.

A key issue in scanning melanoma patients with FDG-PET is whether or not to perform a head-to-toes scan (so-called ‘true’ whole-body imaging). Although no prospective studies have evaluated the value of including the brain and lower extremities in all patients, our current policy is to include the brain and lower extremities only if they are or have been previously, involved (clinically or on other modalities). In the absence of present or past lesions on the lower extremities, we also include them in the imaging field if the patient has proven or suspected disease in the inguino-femoral nodes. Literature review suggests that in the absence of the above factors, the likelihood of finding lesions in the lower extremities is very low (less than 1%) to warrant routine inclusion of these areas in imaging protocols.[86]
